# Exploring the Impact of Single-Nucleotide Polymorphisms on Translation

**DOI:** 10.3389/fgene.2018.00507

**Published:** 2018-10-30

**Authors:** Francis Robert, Jerry Pelletier

**Affiliations:** ^1^Department of Biochemistry, McGill University, Montreal, QC, Canada; ^2^Department of Oncology, McGill University, Montreal, QC, Canada; ^3^Rosalind and Morris Goodman Cancer Research Centre, McGill University, Montreal, QC, Canada

**Keywords:** translation initiation, eIF4F, ribosome recruitment, SNP, genetic variant

## Abstract

Over the past 15 years, sequencing of the human genome and The Cancer Genome Atlas (TCGA) project have led to comprehensive lists of single-nucleotide polymorphisms (SNPs) and gene mutations across a large number of human samples. However, our ability to predict the functional impact of SNPs and mutations on gene expression is still in its infancy. Here, we provide key examples to help understand how mutations present in genes can affect translational output.

## Sequence Variation and Gene Expression

In the last two decades, advances in genome sequencing has provided unprecedented access to the human genome landscape and enabled documentation of sequence variations among individuals. Humans share 99.5% identity at the genomic sequence level implying that the resulting phenotypic diversity stems from the remaining 0.5% difference as well as epigenetic modifications. Sequence differences arise due to the presence of short and variable number tandem repeats, insertion or deletion polymorphisms, and single-nucleotide polymorphisms (SNPs) (Mccarroll et al., 2006; Orr and Chanock, 2008). Among SNPs, transitions (A ↔ G or C ↔ T) are more prevalent than transversions (A ↔ C or T; and G ↔ C or T). There are at least 10 million SNPs within the genome, occurring approximately every 100–300 base pairs and with an allele frequency greater than 1%, making these by far the most common variant type in the human genome (Risch, 2000; Lander et al., 2001; Orr and Chanock, 2008). Recently, there has been a bloom in genome-wide association studies (GWAS) where the prevalence of specific SNPs is linked to phenotypes or disease (Srinivasan et al., 2016). As well, The Cancer Genome Atlas (TCGA) has identified sequence variations between tumor and normal cells and the current challenge is distinguishing between those mutations that exert effects on gene expression to drive tumor evolution versus irrelevant passenger mutations.

Mutations have the potential to alter all steps of gene expression depending on their genomic location. When present within transcriptional regulatory elements, they can affect mRNA expression. When arising in genes, SNPs can impact on mRNA splicing, nucleo-cytoplasmic export, stability, and translation. When present within a coding sequence and leading to an amino acid change (referred to as a non-synonymous SNP or mutation), they can modify the protein’s activity. If the mutation is synonymous (i.e., does not change the nature of the amino acid), then translation rates or mRNA half-life may be affected. If the mutation causes a premature stop codon, this can lead to the production of a truncated protein product or a near-null phenotype due to nonsense mediated decay (Mendell and Dietz, 2001; Nicholson et al., 2010). The Encyclopedia of DNA Elements (ENCODE)^1^ project aims to identify and catalog functional elements in the human genome and has been quite useful in understanding the potential impact that sequence variations exert on gene expression (Consortium, 2012). However, the functional consequences of sequence variants that occur within mRNA 5′ leader [i.e., the region upstream of the initiator codon of the main open reading frame (ORF)] and 3′ untranslated regions (UTRs) (i.e., the region downstream of the major ORF stop codon) are not always immediately obvious and often not characterized. Here, we provide some thoughts on how such variants could affect mRNA translation efficiency. We highlight the individual steps of translation that sequence variants can affect, citing choice examples when appropriate and these are summarized in Table 1.

**Table 1:** Summary of the SNPs described in this study.

Mechanism affected	SNP	Consequence	Reference
Change in mRNA secondary structure	U to C in alanyl tRNA synthetase mRNA	Altered mRNA folding	Shen et al., 1999
	U to C in replication protein A mRNA	Altered mRNA folding	Shen et al., 1999
	G to C in 5′ leader of AASDHPPT	Disruption of a G-quadruplex and translation derepression	Beaudoin and Perreault, 2010
	A to G in coding sequence of COMT mRNA	Altered mRNA folding leading to a change in translational output	Nackley et al., 2006
Start site selection	G to C at position -3 relative to the AUG of BRCA1	Change in optimal Kozak sequence and reduction in translational output	Signori et al., 2001
	A to U at position -3 relative to the AUG of DBI	Change in optimal Kozak sequence and reduction in translational output	Xu et al., 2010
	C to G at position -3 relative to the AUG of PTGS2	Change in optimal Kozak sequence and reduction in translational output	Xu et al., 2010
	G to A at position -26 relative to the AUG of β-globin	Creation of a new AUG out of frame with main AUG that dampens translation at main ORF	Cai et al., 1992
	C to U at position -22 relative to the AUG of GCH1	Creation of a new AUG out of frame with main AUG that dampens translation at main ORF	Armata et al., 2013
	G to U at position -34 relative to the AUG of CDKN2A	Creation of a new AUG out of frame with main AUG that dampens translation at main ORF	Liu et al., 1999; Orlow et al., 2007
Creation of upstream ORFs	G to A at position -75 relative to the AUG of SRY	Creation of a new uORF that dampens translation at main ORF	Poulat et al., 1998; Calvo et al., 2009
	C to U at position -53 relative to the AUG of SPINK1	Creation of a new uORF that dampens translation at main ORF	Witt et al., 2000; Calvo et al., 2009
	G to A at position -420 relative to the main AUG	Creation of a second uORF upstream of the Main AUG; leading to continuous translation under stress	Somers et al., 2015
Loss of upstream ORF	A to G at position +1 of AUG in uORF of EPHB1	uAUG changed to GUG that increased translation at main ORF	Schulz et al., 2018
	U to C at position +2 of AUG in uORF of MAP2K6	uAUG changed to ACG that increased translation at main ORF	Schulz et al., 2018
Mutation in uORF-encoded peptide	G to A in at amino acid 36 of uORF located at position -142 relative to AUG of TGFβ3	Arg to His substitution that increases translation at main ORF	Beffagna et al., 2005
	C to U in coding region of second uORF of HTR3A	Pro to Ser substitution that increases translation at main ORF	Niesler et al., 2001
IRES activity	C to U in the Myc IRES	Increased Myc protein production	Stoneley et al., 1998; Chappell et al., 2000
Alternative splicing	G to C in the splicing donor of intron three of TPO	Shortened 5′ leader where uORF is missing; leads to increased TPO protein	Wiestner et al., 1998
RNA binding protein	C to G at position -22 of AUG in rpS26	Disrupts polypyrimidine tract and decreases translation	Li et al., 2013
Translation elongation rates	C to U at position 3435 of MDR1	Altered protein folding	Kimchi-Sarfaty et al., 2007
	U to G at position 2562 of CFTR	Altered protein folding and reduces protein levels	Kirchner et al., 2017
Mutation in miRNA	G to A in miRNA-1269	Increase SPATS2L and LRP6 protein levels	Min et al., 2017
Mutation in miRNA binding site on mRNA	A to G in the 3′ UTR of TOMM20	Increased levels of TOMM20	Lee et al., 2016


## An Overview of eIF4F-Dependent Ribosome Recruitment

**FIGURE 1:**
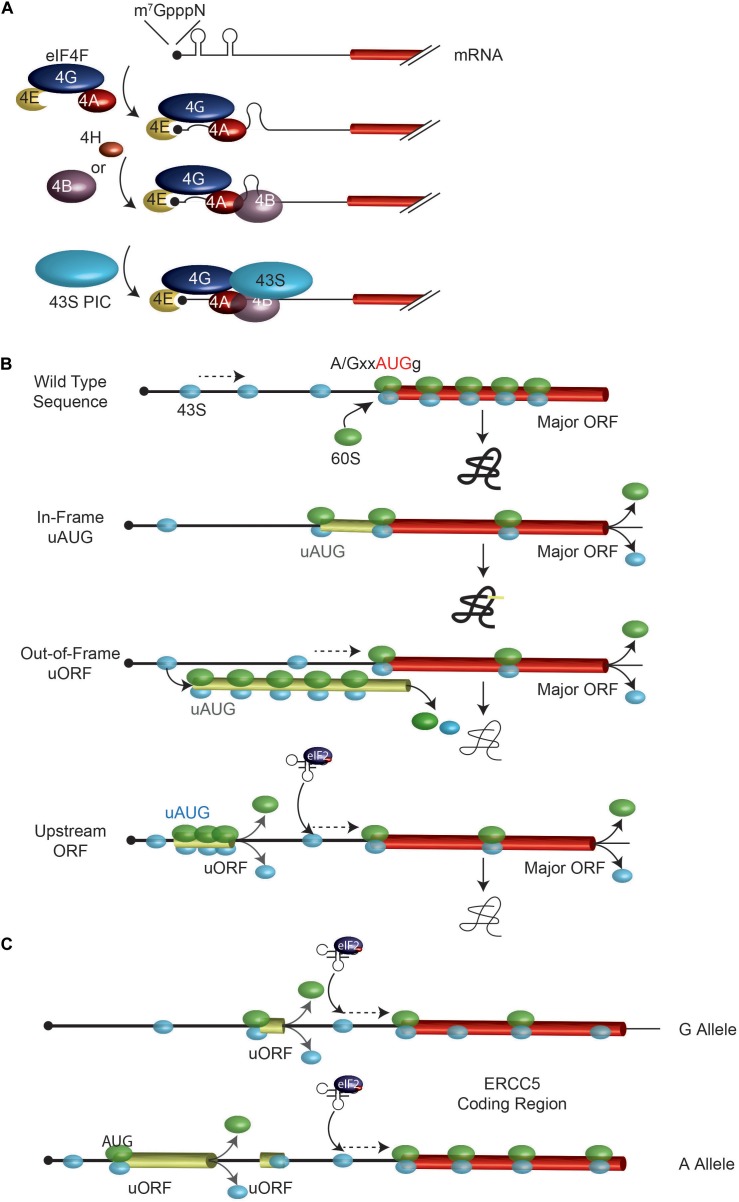
Overview of ribosome recruitment and scanning. **(A)** Cap-dependent translation initiation. The eIF4F complex, in conjunction with eIF4B and eIF4H, serves to prepare the mRNA for 43S ribosomal complex recruitment. **(B)** Impact of uAUGs and uORFs on ribosome scanning. When bound to the mRNA, the 43S PIC (in light blue) scans the mRNA in search for an initiator AUG. An AUG codon in a favorable context is efficiently recognized by the scanning 40S subunit, at which point a 60S subunit will join and elongation begins. Mutations creating novel uAUGs or uORFs will influence the frequency of ribosomes that initiate at the major ORF AUG codon. The position of an uORF, relative to the major AUG codon is important in determining major AUG utilization since the distance from the uORF stop codon and the major AUG dictates the time it will take for a ribosome to re-acquire a eIF2^∗^GTP^∗^Met-tRNA ternary complex. **(C)** A G/A SNP in the *ERCC5* mRNA 5′ leader region controls expression and response to stress. The A allele containing mRNA has an additional uORF which allows for more efficient *ERCC5* main ORF translation under situations when eIF2α is phosphorylated. See text for details.

Mammalian protein synthesis is predominantly regulated at the step of translation initiation, with the rate-limiting step being the recruitment of ribosomes to mRNAs (Figure 1A; Sonenberg and Hinnebusch, 2009). The key mediator of this step is the eIF4F complex. The eIF4E subunit binds to the mRNA cap structure present on all eukaryotic cytoplasmic mRNAs. The eIF4G component has RNA binding domains and stabilizes the eIF4E: cap interaction (Marcotrigiano et al., 2001; Yanagiya et al., 2009). RNA structural elements are resolved by the eIF4A DEAD-box RNA helicase in conjunction with RNA binding proteins, eIF4B and/or eIF4H (Figure 1A). The 43S pre-initiation complex (40S ribosome and associated factors) (PIC) is then recruited to the mRNA template via bridging interactions between eIF4G and ribosome-bound eIF3 (Sonenberg and Hinnebusch, 2009). This mode of initiation is referred to as cap- or eIF4E-dependent. The requirement for eIF4F by mRNAs to recruit ribosomes differs and appears to scale as a consequence of 5′ leader secondary structure (Pickering and Willis, 2005; Bitterman and Polunovsky, 2015; Hinnebusch et al., 2016). Since eIF4E is thought to be limiting for translation, mRNAs must compete for access to eIF4F and those with structural barriers in their 5′ leader region are at a disadvantage (Pelletier and Sonenberg, 1985; Babendure et al., 2006). Hence, altering the secondary structure landscape within the mRNA 5′ leader region can significantly impact on translational efficiency by affecting ribosome recruitment rates (Pelletier et al., 2015). Once bound, the 43S PIC scans the mRNA 5′ leader region in search of an initiation codon.

A second mechanism by which the 43S PIC can be recruited to mRNA templates is through direct internal binding within the 5′ leader region to an internal ribosome entry site (IRES), obviating the requirement for the cap structure. Best characterized among these are the viral IRESes and these have been stratified in four classes, based on structural similarities, initiation factor and/or IRES trans-acting factor requirements (Mailliot and Martin, 2018). Some exceptional IRESes, such as the cricket paralysis virus IRES, bypass the need for any initiation factors and can directly bind to the ribosome.

The discovery and characterization of IRESes in cellular mRNAs is of significant interest since they have been implicated in allowing translation to proceed under conditions when cap-dependent translation is impaired, such as stress, apoptosis, nutrient limitation, and mitosis (Komar and Hatzoglou, 2015). Cellular IRES function is therefore thought to be important for allowing rapid adaptation to a quickly changing environment, with selective translational effects being the outcome. Influences of SNPs on cellular IRESes activity could affect response to stress such as hypoxia, heat shock, toxins, or drugs (chemotherapy). As well, mutations in cellular IRESes could lead to aberrant translational responses to drive a number of pathological disorders, ranging from autoimmune disease, neurodegeneration, and cancer (Holcik and Sonenberg, 2005).

## Changes in Secondary Structure Affecting Translational Output

By impeding eIF4F-cap interactions or ribosome scanning, structural features (e.g., stem-loops, RNA-protein complexes, G-quadruplexes) can act as barriers to translation initiation and negatively impact translational efficiency (Pelletier and Sonenberg, 1985; Babendure et al., 2006). A study by Shen et al. (1999) was one of the first to document the extensive impact that SNPs can have on mRNA secondary structure (Table 1). Analysis of two SNPs within the coding regions of mRNAs encoding alanyl tRNA synthetase and replication protein A uncovered allele-specific structural features that impacted on sequence accessibility. Evidence that such changes can affect translational output was provided by a study assessing the influence of G-quadruplex structures present in 5′ leader regions on translation (Beaudoin and Perreault, 2010). A SNP (G to C change) was identified at position 7 of a G-quadruplex (a critical region for G-quadruplex formation) within the 5′ leader of the *AASDHPPT* (aminoadipate-semialdehyde dehydrogenase-phosphopantetheinyl transferase) mRNA (Beaudoin and Perreault, 2010). Biophysical experiments showed that G-quadruplex formation was disrupted and this was associated with 1.5-fold increase in protein levels in cells, with no effect on mRNA levels (Beaudoin and Perreault, 2010). These experiments indicate that point mutations in 5′ leader regions that alter secondary structure can impact on translational output.

Secondary structure immediately downstream of the AUG can also affect translational output. For example, an A to G synonymous SNP at a *Leu* codon, present in the coding region of the catechol-*O*-methyltransferase (*COMT*) mRNA, was identified in subjects with high pain sensitivity and at greater risk of developing temporomandibular joint disorder (Diatchenko et al., 2005; Nackley et al., 2006). The *COMT* protein is responsible for catecholamine degradation and is a regulator of pain perception. In humans, three major haplotypes are formed by four SNPs at the *COMT* locus: one located in the promoter and three in the coding region [two synonymous at *his^62^his* (C/T) and *leu^136^leu* (C/G) and one non-synonymous *val^158^met* (A/G)] (Nackley et al., 2006). It was reported that the major *COMT* haplotypes varied with respect to local mRNA secondary structure with the most stable structure associated with the lowest levels of protein production (Nackley et al., 2006). Site-directed mutagenesis disrupting the structural element caused an increase in protein production. The authors did not, however, directly assess the effect of the different haplotype sequences on mRNA translation rates.

Conversely, secondary structure can also act to increase start codon recognition when appropriately located downstream of initiation codons – an effect presumably due to the slowing of scanning ribosomes and increased codon sampling time (Kozak, 1991). Hence, sequence changes that increase the formation of structure in the AUG downstream proximal region could increase AUG utilization rates.

## 5′ Leader Sequence Variation and Start Site Selection

### SNPs Affect Start Codon Recognition

The mechanism by which 43S PICs locate an initiation codon has consequences on how SNPs that generate new, or remove existing, start codons affect translation initiation. The sequence context of an initiation codon dictates the efficiency by which it is recognized by scanning 43S PICs (Figure 1B). The optimal context is A/GxxAUGg, referred to as the Kozak consensus sequence, with the -3 purine (relative to the A of the AUG) being the most important determinant (Kozak, 1986, 1987a). Mutations that change this context are predicted to affect start site selection efficiency.

There are many examples of mutations that alter the AUG sequence context to impact on translational efficiency. One such example is the description of a mutation within the BRCA1 gene in a 35 years old patient converting a G to C at the -3 position relative to the BRCA1 AUG initiation codon (Signori et al., 2001). This mutation changes an optimal purine to a less favorable pyrimidine and has been linked to sporadic breast and ovarian cancers (Hall et al., 1990; Szabo and King, 1995). *In vitro* and *in vivo* expression studies of reporter mRNAs harboring the C allele showed a 30–50% decrease in protein expression compared to control mRNAs harboring the G allele. As well, the transcript carrying the G allele was associated with heavier polysomes (and hence elevated translation rates) compared to the C allele containing mRNA.

The NCBI SNP database has been mined for the presence of variants spanning AUG initiation codons, with a focus on the -3 and +4 positions (Xu et al., 2010). This study identified SNPs in >45 genes that occurred at one of these two critical positions and could thus potentially affect AUG utilization. The variants of two genes were tested by transfection of reporter constructs into cells and revealed that mRNAs harboring SNPs with “weaker” or “stronger” Kozak consensus sequence produced reduced or elevated protein levels, respectively (Xu et al., 2010). No differences in mRNA levels were noted.

### SNPs Creating an In-Frame uAUG

Mutations that generate start codons upstream, and in frame with, the major initiation codon of an open reading frame will “catch” some scanning 43S PICs and redirect protein synthesis to the new start site to produce N-terminal extended protein products (Figure 1B, see “In-frame uAUG”). The efficiency with which this is achieved is dictated, in part, by the context surrounding the new initiation codon.

One bioinformatics tool which has been developed for categorizing effects of variants on genome function is SnpEff (Cingolani et al., 2012). This tool annotates variants based on genomic locations to include intronic, untranslated region, splice site, intergenic, non-synonymous coding, etc… (Cingolani et al., 2012). Among the effects listed by SnpEff are changes in initiation codons (AUG and the less common CUG and UUG codons) that occur in the 5′ leader region. Of 297 SNPs that generated a new translation initiation codon in the 5′ leader region when comparing two *Drosophila melanogaster* strains, ∼25% were in the same reading frame as the major ORF (Cingolani et al., 2012) and would produce N-terminally extended polypeptides.

### SNPs Creating an uORF Out-of-Frame With the Major ORF

If a mutation generates a new start codon out-of-frame with the major ORF AUG, some 43S PICs may initiate at the new upstream (u) ORF and by-pass the major ORF, resulting in a decrease in protein production from the major ORF-encoded product (Figure 1B and Table 1). The extent of re-routing will depend, in part, on the context of the novel initiation codon as well as AUG proximal secondary structure (Kozak, 1991; Barbosa et al., 2013).

As an example of such a scenario, a germline mutation in the β-globin gene 26 nt upstream of the initiator AUG codon leads to the creation of a new, out-of-frame uAUG (Oner et al., 1991; Cai et al., 1992). This uAUG is in a favorable sequence context (A in the -3 position) and initiation at this uAUG shunts ribosomes pass the authentic AUG, reducing β-globin production, and leading to β-thalassemia. Whether or not mRNA stability is affected by a particular 5′ leader mutation and this also contributes to the phenotype needs to be carefully assessed.

A similar scenario has been documented in the GTP cyclohydrolase 1 gene (*GCH1*) in which heterozygous mutations are associated with Dopa-responsive dystonia (DRD) (Armata et al., 2013). Here, a germline C to T transition 22 nt upstream of the translation start site generates a novel start codon that is out-of-frame with the downstream *GCH1* AUG codon and results in reduced GCH1 production (Armata et al., 2013). It will be important to extend these results to: (i) formally demonstrate that the C to T transition leads to translation of the newly created uORF (an assessment that can be made by ribosome footprinting) and (ii) demonstrate that the C to T alteration leads to changes on endogenous GCH1 protein output.

The impact that this class of mutations can have on tumor biology is significant and is exemplified by the identification of a germline mutation in the *CDKN2A* tumor suppressor gene mapping 34 nucleotides upstream of the normal start site (Liu et al., 1999; Orlow et al., 2007). In this case, a G to T mutation creates a novel initiation codon residing in a favorable context but out-of-frame with the *CDKN2A* AUG. The T allele thus generates an mRNA that produces less *CDKN2A* and substantially increases the risk of melanoma in carriers (Liu et al., 1999; Orlow et al., 2007).

### SNPs Creating an uORF Upstream of the Major ORF

If the presence of a SNP leads to creation of a new uORF, this may impact gene expression by: (i) affecting re-initiation efficiency at the downstream major ORF, and (ii) generating a novel peptide encoded by the uORF (Barbosa et al., 2013). The precise mechanism of how 40S ribosomes are able to resume protein synthesis after having translated an uORF is not well defined but is related to the length of the uORF (the longer the uORF, the less efficient the re-initiation process) as well as the presence of structural barriers in the uORF (which reduces re-initiation potential) (Figure 1B; Kozak, 1987b; Abastado et al., 1991; Poyry et al., 2004). It is thought that initiation factors critical for re-initiation remain ribosome-bound for some time following commencement of elongation, but at some point are lost or ejected from the translating ribosome. If termination of translation occurs before these factors are lost, then that ribosome maintains its ability to reinitiate (Poyry et al., 2004). An analysis of 11,649 matched mRNA and protein measurements from four published mammalian studies have indicated that the presence of uORFs within the 5′ leader region is generally associated with reduced expression from the major ORF (Calvo et al., 2009).

The repressive nature of a newly created uORF can, in part, stem from the reduced efficiency associated with translation re-initiation compared to *de novo*, cap-dependent translation initiation. Calvo et al. (2009) undertook a search for ORF-altering nucleotide variants within 12 million SNPs present in dbSNP^2^. They found a number of novel and previously described polymorphisms predicted to create new, or remove existing, uORFs. For example, mutations within the 5′ leader region of the *SRY* (Poulat et al., 1998) and *SPINK1* (Witt et al., 2000) mRNAs introduced novel uORFs upstream of the major ORF. Testing of reporters with different 5′ leaders showed that those harboring uORFs produce less major ORF protein compared to reporters expressing control, wild-type 5′ leader sequences. In general, the occurrence of a new uORF is associated with a 30–80% decrease in protein synthesis from the major ORF (Calvo et al., 2009).

Mutations that lead to the loss of an uORF can increase translation output. Analysis of 404 uORFs present in the 5′ leaders of mRNAs encoding 83 tyrosine kinases and 49 other proto-oncogenes in 308 human malignancies uncovered uORF mutations in the *EPHB1* and *MAP2K6* genes (Schulz et al., 2018). In the case of *EPHB1*, a mutation changed the only uAUG found in the 5′ leader to a GUG codon, while the sole uAUG of *MAP2K6* was modified to an ACG codon. Both of the identified mutations lead to an increase in translational output from their respective mRNAs. This was complemented by a computational analysis of whole exome sequencing data from 464 colon cancers which revealed somatic mutations leading to the loss of 22 uORF initiation and 31 uORF termination codons (Schulz et al., 2018).

The presence of an uORF has also been shown to confer resistance to cisplatin exposure by facilitating translation of the major ORF encoded polypeptide under stress conditions (Somers et al., 2015; Figure 1C). The *ERCC5* gene encodes an endonuclease that cleaves 3′ of DNA adducts and is required for nucleotide excision repair. The mRNA 5′ leader region harbors an uORF. There is a G/A polymorphism, rs751402, located upstream of this uORF where the A allele containing mRNA has a novel uORF, but the G allele containing mRNA does not. Treatment of cells with cisplatin leads to induction of a stress response, resulting in phosphorylation of eIF2α and a longer persistence in translation of the A allele mRNA (Somers et al., 2015). Whereas eIF2α phosphorylation is generally associated with a global shut down of protein synthesis, the translational output from some mRNAs is paradoxically increased due to the uORF configuration within their 5′ leader regions.

eIF2 is required for ternary complex formation (with tRNA and GTP). When the eIF2α subunit becomes phosphorylated, ternary complex formation becomes rate-limiting resulting in a global shut down of general translation. Ribosome re-initiation following the translation of an uORF must recruit *de novo* ternary complexes and increasing the distance of the uORF to the next downstream AUG codon allows more time for that event to take place (Figure 1C). In the case of the *ERCC5* A allele-containing mRNA, the creation of an uORF makes it such that under stress, most ribosomes that have completed translation of the A-encoded uORF will not re-acquire another ternary complex before having scanned past uORF2 (and hence uORF2 won’t be translated), but will do so before reaching the *ERCC5* ORF (Figure 1C). The creation of new uORFs and their location within the 5′ leader region can thus alter how translation of specific mRNAs respond to signaling cues.

### SNPs Affecting an uORF Coding Region

Mutations arising within the coding region of uORFs have the potential to exert two types of effects on translation – by affecting the nature of an encoded regulatory peptide and by altering elongation rates.

If they perturb the function of a regulatory peptide involved in dictating ribosome re-initiation rates, then they can affect the output from the major ORF. Such might be the case for a G/A SNP in the 5′ leader of the transforming growth factor β3 (*TGFβ3*) mRNA and present in several members of a large pedigree with arrhythmogenic right ventricular cardiomyopathy type 1 (Beffagna et al., 2005). The *TGFβ3* mRNA 5′ leader contains 11 AUGs potentially encoding 11 polypeptides (Arrick et al., 1991). The G/A SNP does not alter uORF configuration but rather causes an Arg to His substitution at codon 36 of an 88 amino acid uORF that is out of frame and overlaps with the sequence of the *TGFβ3* main AUG. When tested in the context of a luciferase reporter assay in transfected C2C12 myoblasts cells, the presence of the A variant lead to a 2.5-fold increase in luciferase production (Beffagna et al., 2005). A similar situation was reported for the serotonin receptor gene, *HTR3A*, where a C/T SNP located in the second uORF caused a Pro to Ser change in individuals with bipolar affective disorder (Niesler et al., 2001). The authors tested the consequences of this SNP in a luciferase-based transfection assay and found that the T allele caused a 2.5- to 2.9-fold increase in luciferase expression without altering mRNA levels. One interpretation of these results is that the uORF-encoded peptide plays an inhibitory role in translation and the G to A change impairs activity of this small polypeptide. In both the aforementioned studies, potential effects of the SNP on splicing need to be assessed to rule out other possible explanations for the observed effects.

Alternatively, if variants influence uORF elongation rates then they can influence the potential for re-initiation at downstream AUG codons (Jackson et al., 2012; Gunisova et al., 2018). Slowing down elongation rates of ribosomes transiting the uORF is thought to increase the probability that initiation factors associated with elongating ribosomes, and necessary for elongation, will be released before completion of uORF translation. This would then lead to decreased re-initiation at downstream AUG codons. Conversely, if elongation rates within the uORF are increased, this might lead to increased re-initiation rates and protein production from downstream ORFs.

## 5′ SNPs and IRES Activity

Another manner by which sequence changes within 5′ leader regions have been documented to alter translation is by affecting IRES activity. The *c-Myc* (*MYC*) proto oncogene has been reported to harbor an IRES which may contribute to translation mis-regulation of *MYC* during tumorigenesis (Stoneley et al., 1998). Interestingly, a point mutation within the *MYC* 5′ leader region leading to a C to T transition was identified in a multiple myeloma cell line and associated with elevated *MYC* protein levels (Paulin et al., 1998). The 5′ leader harboring the T allele showed enhanced binding of several RNA binding proteins, as revealed by Northwestern blotting and UV crosslinking approaches. The same C to T mutation was found in 42% of primary multiple myeloma samples and generated an IRES variant that appeared to be more active (Chappell et al., 2000). The underlying mechanism for how the C/T change can lead to alterations in IRES activity awaits further definition.

## 5′ SNPs, Transcription Initiation Site Selection, and Alternative Splicing

Single-nucleotide polymorphisms present within gene regulatory regions can affect transcription factor, as well as RNA Pol II binding (Kasowski et al., 2010). If RNA Pol II binding is redirected to a newly formed site, this could lead to usage of alternative transcription initiation sites – generating mRNAs with differing 5′ leader sequences and which could affect translation initiation rates.

Sequence variation in the 5′ leader region can also occur through alternative mRNA splicing to produce isoforms with different translational efficiency. The presence of SNPs that impact on alternative splicing can change the levels and nature of the resulting mRNA isoforms. For example, thrombopoietin (*TPO*) is a master regulator of megakaryopoiesis and platelet production and is under tight translational control. Its 5′ leader has seven uORFs (Ghilardi et al., 1998). A SNP has been identified that increases *TPO* serum levels in patients with hereditary thrombocythaemia (Wiestner et al., 1998), a genetic disorder caused by elevated platelet levels due to sustained proliferation of megakaryocytes (Murphy et al., 1997). Specifically, a G → C transversion at the splicing donor site of *TPO* intron 3 is responsible for generating a shortened 5′ leader where uORF 7, as well as the main AUG, is lost (Wiestner et al., 1998). Translation initiation thus occurs at the next downstream AUG and leads to a fully functional, although truncated, *TPO* protein product where levels produced are much higher than from the normal mRNA. This effect appears to be the result of increased translation, presumably through effects on the re-initiation process, since the SNP did not affect RNA levels (Wiestner et al., 1998). Whether SNPs affect splicing or transcription, can only be assessed through sequence characterization of mRNA 5′ leader regions, an analysis that is all too frequently omitted.

## 5′ SNPs and RNA Binding Protein Target Sites?

Impacting on RNA binding protein target sites is another manner by which SNPs could affect translation. By measuring the ratio of polysome- to monosome-bound mRNAs in immortalized lymphoblastoid cell lines, a genome-wide search for SNPs affecting translational efficiency was undertaken (Li et al., 2013). This study found that a SNP within the 5′ leader region of the small ribosomal protein S26 mRNA (rs1131017: C/G located 22 nucleotides upstream of the initiator AUG codon) was associated with altered protein production. Reporter mRNAs harboring the G variant produced more protein than mRNAs having the C variant. This SNP is in high linkage disequilibrium with the 12q13 locus for susceptibility to type I diabetes. It interrupts a polypyrimidine track (….^-28^TCTCCT[C/G]TCTCC^-17^…) upstream of the rpS26 AUG codon. Whether this alters the binding of an RNA binding protein, such as polypyrimidine tract-binding protein (which has been implicated in translation initiation), remains to be determined (Kaminski and Jackson, 1998).

## SNPs and Elongation Rates

The information contained within mRNAs that encode the proteome is encrypted by 61 possible codons. Codons encoding the same amino acid are decoded by cognate tRNAs, which are not equally expressed in cells. It is generally thought that codon decoding rates can vary as a function of tRNA abundance and this can have dramatic effects on elongation rates (Cannarozzi et al., 2010; Hanson and Coller, 2018). This has been borne out by ribosome footprinting data and by experiments where translational output has been increased simply by replacing rare codons with more frequent ones (Gardin et al., 2014; Lareau et al., 2014; Hussmann et al., 2015; Weinberg et al., 2016). However, rare codons are thought to play important roles in cellular homeostasis since stretches of rare codons induce ribosome pausing during elongation and this provides time for proper protein folding (Hanson and Coller, 2018). Thus, a SNP changing a rare codon to a more common one could, in principle, increase protein output but decrease the proportion of functional (i.e., correctly folded product) polypeptide synthesized.

An example where codon usage could affect protein activity is exemplified by a study assessing the impact of a synonymous SNP (C3435T) present within the multidrug resistance 1 (*MDR1*) coding region on protein function (Kimchi-Sarfaty et al., 2007). The *MDR1* gene encodes an ATP-driven drug efflux pump that contributes to drug resistance in tumor cells. The C3435T SNP had been previously associated with reduced *MDR1* expression and function in human cells (Hoffmeyer et al., 2000; Drescher et al., 2002). This SNP changes the most common Ile codon (AUC) to a less prevalent one (AUU). Reporter constructs harboring the C or T variants show similar protein expression levels, but produce products with different activity (Kimchi-Sarfaty et al., 2007). Trypsin digestion experiments revealed that the *MDR1* product from the two different haplotypes differ in their protease sensitivities indicating distinct conformations. Conversion of the Ile codon to an ever rarer one, AUA, generated an mRNA that produced MDR1 protein with even less drug transport activity.

A similar phenomenon was observed for the cystic fibrosis conductance transmembrane regulator (*CFTR*) gene, in which a T2562G synonymous SNP in the coding region was found to reduce protein levels by 30% without affecting mRNA levels or splicing (Kirchner et al., 2017). This SNP changed a threonine codon from the highly prevalent ACU sequence to the rarer ACG codon. A *CFTR* expression vector bearing the G allele showed reduced single-channel Cl^-^ conductance function compared to a T allele expressing vector (Kirchner et al., 2017). The authors concluded that slower synthesis rate from the G allele encoded mRNA resulted in improper protein folding that targeted *CFTR* for degradation by the quality-control machinery (Kirchner et al., 2017). The reduced protein levels from the G allele mRNA were rescued by transfection of an expression vector driving synthesis of the SNP-corresponding cognate tRNA (Kirchner et al., 2017).

## Sequence Variation in 3′ Utrs Affecting Translation

With the exception of histone mRNAs, cellular mRNAs have poly (A) tails at their 3′ end. The poly (A) tail is important for translation initiation and its function is mediated by the poly(A) binding protein, PABPC1. PABPC1 also interacts with eIF4G at the 5′ end of the mRNA to create an mRNA closed loop that is thought to stimulate translation by: (i) stabilizing the association of eIF4F with the cap, (ii) stimulating 60S ribosomal subunit binding, and (iii) increasing the effective concentration of terminating ribosomes in proximity of the cap structure. SNPs that mutate the polyadenylation signal will lead to the generation of isoforms with longer 3′ ends due to usage of downstream polyadenylation sites (Thomas and Saetrom, 2012). If the extended sequence results in the acquisition of novel microRNA (miRNAs) binding sites, then regulation of the new mRNA isoform can be quite different than the wild-type mRNA (Sandberg et al., 2008).

As well, mutations that occur within miRNA target sites and alter miRNA recognition can exert effects on mRNA expression through reduced translation initiation and increased mRNA degradation (Mohr and Mott, 2015). The last decade has seen an extensive list of SNPs that map to miRNAs or their putative binding sites within mRNAs that could potentially affect miRNA response and these have been comprehensively reviewed (Detassis et al., 2017; Moszynska et al., 2017). For example, a G to A SNP has been described in miR-1269, a miRNA linked to increased risks of hepatocellular carcinoma (Min et al., 2017). *SPATS2L* and *LRP6* encode for pro-oncogenic activities and are both targets of miR-1269. This study showed that when the miR-1269 A variant is expressed in cells, the repressive effect on *SPATS2L* and *LRP6* is diminished, compared to the miR-1269 G variant. SNPs in microRNA target sites on mRNAs have also been documented. For example, an A to G SNP in the 3′ UTR of *TOMM20* mRNA was found to be associated with greater risks of colorectal cancer (Lee et al., 2016). The microRNA miR-4273-5p was identified as being responsible for controlling the levels of *TOMM20*.

There are several examples of 3′ UTR RNA binding proteins that can affect mRNA translation; both at the initiation and elongation steps (Szostak and Gebauer, 2013; Yamashita and Takeuchi, 2017). The best example is 4EHP (also known as eIF4E2), a cap binding protein known to also interact with specific mRNA-bound proteins present within the mRNA 3′ UTR. 4E-HP thus forms a closed-loop structure and since it does not interact with eIF4G, this prevents ribosomes from being recruited to the cap structure and exerts mRNA-selective inhibition of translation (Morita et al., 2012; Szostak and Gebauer, 2013; Chapat et al., 2017; Yamashita and Takeuchi, 2017). SNPs affecting RNA binding proteins that interact with 4EHP could lead to alterations in expression of a specific set of 4EHP-responsive mRNAs.

## Conclusion

Whereas significant effort has been placed on finding and annotating SNPs that can affect protein function using programs such as SIFT (Kumar et al., 2009) and PolyPhen (Adzhubei et al., 2010; Li and Wei, 2015), there is a recognized need for bioinformatics tool that can predict potential functional consequences of SNPs in mRNA 5′ leader and 3′ UTRs (Kumar et al., 2014). Advances have been made (i) regarding software that predicts the effects of SNPs on miRNA targets, with programs such as microSNiPer (Barenboim et al., 2010) and mrSNP (Deveci et al., 2014), (ii) identification of translation initiation sites using ATGpr, and (iii) ORF prediction software such as ORF Finder. What is now needed are tools like SnpEff that could link changes in 5′ leader and 3′ UTR sequences to predictions on major ORF expression. A better understanding of the variables involved in determining mRNA translation efficiency will help design algorithms with more quantitative predictive power.

Much has been learnt from the functional analysis of genetic variants within mRNA 5′ leaders and their effects on translation. The majority of these were identified because they were associated with an observable phenotype. The lesions whose effects are easiest to predict are those affecting initiation codon context or leading to the generation of novel uAUGs. However, it is those whose effects remain unexplained that will likely lead to the uncovering of new biological mechanisms. For example, during a search for oncogenic changes associated with prostate cancer, Wang et al. (2009) identified a G to A somatic mutation that mapped within the δ-catenin 5′ leader region, nine nucleotides upstream of the AUG codon. The presence of the A allele in reporter mRNAs resulted in a threefold to sevenfold increase in protein expression relative to mRNAs harboring the G allele, with no effect on mRNA levels noted. The mechanism underlying this translational stimulation is unknown but points to some very interesting biology. It also underscores the need to carefully consider the functional consequence of 5′ leader mutations uncovered by large scale cancer genome sequencing projects and their potential role in affecting translational output. This is currently difficult to do systematically due to deficiencies in our ability to predict RNA structural complexity, as well as a lack of knowledge on the RNA binding protein (RBP) landscape *in vivo*. Genome-wide RNA structure probing approaches, as well as efforts aiming to define the RBP interactome, are being undertaken to fill this void (Castello et al., 2013; Tenzer et al., 2013; Bevilacqua et al., 2016; Bisogno and Keene, 2018).

## Author Contributions

All authors drafted and wrote the review.

## Conflict of Interest Statement

The authors declare that the research was conducted in the absence of any commercial or financial relationships that could be construed as a potential conflict of interest.

## Footnotes

1 https://www.encodeproject.org/

2 https://www.ncbi.nlm.nih.gov/projects/SNP/
